# Reduced expression of lncRNA *DLEU7-AS1* is a novel favorable prognostic factor in acute myeloid leukemia

**DOI:** 10.1042/BSR20212078

**Published:** 2022-05-18

**Authors:** Cui-zhu Wang, Bei-bei Ma, Zi-jun Xu, Jing-dong Zhou, Ting-juan Zhang, Qin Chen, Dong-ming Yao, Jiang Lin, Jun Qian, Suo Sha

**Affiliations:** 1 Department of Oncology, Affiliated Haian Hospital of Nantong University, Nantong, Jiangsu, People’s Republic of China; 2 Cardiac Intensive Care Unit, Affiliated Hospital of Jining Medical University, Jining, Shandong, People’s Republic of China; 3 Laboratory Center, Affiliated People’s Hospital of Jiangsu University, Zhenjiang, Jiangsu, People’s Republic of China; 4 The Key Lab of Precision Diagnosis and Treatment of Zhenjiang City, Zhenjiang, Jiangsu, People’s Republic of China; 5 Department of Hematology, Affiliated People’s Hospital of Jiangsu University, Zhenjiang, Jiangsu, People’s Republic of China; 6 Surgery of Traditional Chinese Medicine, Haian Hospital of Traditional Chinese Medicine, Nantong, Jiangsu, People’s Republic of China

**Keywords:** AML, DLEU7-AS1, expression, karyotype

## Abstract

The objective of our study was to measure DLEU7-AS1 expression in *de novo* acute myeloid leukemia (AML) whilst also analyzing its clinical relevance. We used gene expression data from The Cancer Genome Atlas (TCGA), Gene Expression Omnibus (GEO), Cancer Cell Line Encyclopedia (CCLE) and Genotype-Tissue Expression project (GTEx) to assess the expression profile of DLEU7-AS1 in pan-cancers, cancer cell lines and normal tissues. Reverse transcription-quantitative PCR was used to measure DLEU7-AS1 expression in bone marrow from 30 normal individuals and 110 patients with *de novo* AML. DLEU7-AS1 expression was found to be markedly reduced in the AML samples of the TCGA pan-cancer datasets. In our PCR validation, DLEU7-AS1 expression was significantly decreased in the AML samples compared with that in controls (*P*<0.001). Low DLEU7-AS1 expression (DLEU7-AS1^low^) correlated positively with lower blood platelet counts (*P*=0.029). In addition, low DLEU7-AS1 expression was more frequently observed in the intermediate (58%; 44/76) and favorable karyotypes (65%; 15/23) compared with that in the poor karyotype (10%; 1/10; *P*=0.005). In particular, patients with high expression levels of DLEU7-AS1 (DLEU7-AS1^high^) showed lower complete remission rates (*P*=0.002) than patients with DLEU7-AS1^low^. Survival analysis revealed that patients with DLEU7-AS1^low^ had longer overall survival (OS) than patients with DLEU7-AS1^high^ (*P*<0.05). Multivariate Cox analysis demonstrated that in patients with non-acute promyelocytic leukemia (non-M3) who were ≤60 years old, DLEU7-AS1 expression was an independent prognostic factor for OS. Furthermore, we found distinct correlations among the expression of DLEU7-AS1, infiltration by immune cells and immune checkpoint genes in AML.

## Introduction

As an important group of common hematopoietic and highly heterogeneous malignancies, acute myeloid leukemia (AML) is characterized by distinct chromosomal and genetic abnormalities, which ultimately leads to the accumulation of anomalous myeloblasts. Although the prognostic importance of its high level of molecular heterogeneity has been well established over the past two decades, translation of this novel information into therapeutic strategy improvements and optimization remains at a primitive stage. The prognosis for the majority of AML subtypes remains dismal due to a lack of effective diagnostic methods and optimal long-term therapeutic regimens [1–3]. It has been frequently reported that the typical AML phenotype results from complex epigenetic/genetic aberrations, which affects cell proliferation, differentiation and apoptosis [4,5]. In particular, it is now becoming clear that cytogenetically normal AML (CN-AML) is highly heterogeneous on molecular levels according to the 2017 World Health Organization (WHO) classification of AML [6]. Cytogenetic alterations, combined with mutations in *nucleophosmin 1* (*NPM1*), *CCAAT/enhancer-binding protein α* (*CEBPA*), *fms-like tyrosine kinase 3- internal tandem duplication* (*FLT3-ITD*) *and TP53*, have been incorporated into the current prognostic grading system for patients with AML [7]. In addition, the overexpression of *brain and acute leukemia cytoplasmic protein* (*BAALC*), *Echovirus 11* (*EVI1*) and *E-26 transformation-specific-related gene* (*ERG*) has also been reported to be beneficial for outcome prediction with high accuracy [7]. Nevertheless, it remains of key importance to identify a novel set of integrated AML-related biomarkers that can have profound impacts on diagnosis, prognosis and individual treatment.

Long non-coding RNAs (lncRNAs) are non-coding RNAs that are >200 nucleotides in length and belong to an emerging category of RNAs that can serve key roles in various types of cancer [8]. Due to their tumor-specific expression patterns, lncRNAs may either serve as tumor suppressors or oncogenes, rendering them important components in the field of cancer research [9]. Previous studies have found that aberrant expression or mutation of lncRNAs can not only increase susceptibility to cancer development but also promote tumorigenesis and metastasis by regulating the expression of related protein-coding genes on different transcriptional levels [10]. As a result of next-generation sequencing technologies, large quantities of novel, distinct lncRNAs have been discovered to be abnormally expressed or mutated in a variety of cancers [11,12]. However, the majority of these lncRNAs have yet to be annotated [11,12]. Characterizing these lncRNAs in addition to understanding the mechanism underlying their function may assist in improving diagnosis and highlight novel therapeutic targets.

The lncRNA deleted in lymphocytic leukemia 7-antisense RNA 1 (DLEU1), originating from 13q.14.3 region, has been previously identified as an up-regulated oncogenic lncRNA in colorectal cancer (CRC). Following the knockdown of DLEU7-AS1 in CRC cell lines HT-29 and HCT-116, the expression levels of key proteins in the Wnt/β-catenin pathway β-catenin, c-Myc and cyclin D1 were all found to be markedly reduced [13]. This suggests that DLEU7-AS1 may promote the occurrence and development of CRC by modulating the Wnt/β-catenin signaling pathway [13]. However, the DLEU7-AS1 expression profile in hematopoietic malignancies remains poorly understood. Therefore, in the present study, we aimed to measure DLEU7-AS1 expression in patients with AML and analyze its relevant clinical significance. Using bioinformatics analysis, we also analyzed the correlation between the expression levels of DLEU7-AS1 with immunocyte infiltration and immune checkpoint genes in AML, in addition to the potential biological functions of DLEU7-AS1.

## Materials and methods

### Patients and bone marrow samples

Bone marrow (BM) specimens were collected from a total of 30 healthy donors and 110 patients with AML. BM mononuclear cells (BMMNCs) were extracted by gradient centrifugation and lymphocyte separation medium (TBD Sciences, Tianjin, People’s Republic of China). According to the 2016 WHO criteria and French-American-British (FAB) classification system [14,15], all patients with *de novo* AML enrolled had a detailed diagnosis. Karyotypes were analyzed at first diagnosis using the conventional R-banding method whereas karyotype risk was classified into three groups (favorable, intermediate or poor) according to the 2017 European leukemiaNET (ELN) risk stratification by genetics [3]. In addition to cytogenetics, ELN 2017 assesses the risk of patients by taking into consideration the effects of mutations in certain genes, such as *NPM1*, *CEBPA*, *FLT3*-ITD, *runt-related transcription factor 1*, *additional sex-combs like 1* and *TP53* [3]. The detailed treatment information and inclusion criteria were kept consistent with our previously published reports [16,17]. Briefly, all patients with AML included in this study received chemotherapy, including induction therapy and subsequent consolidation treatment. Induction therapy for non-M3 AML consists of two courses of daunorubicin combined with cytarabine. Subsequent consolidation treatment included high-dose cytarabine, mitoxantrone with cytarabine and homoharringtonine combined with cytarabine. By contrast, patients with M3 were treated with all-trans retinoic acid (ATRA) together with daunorubicin in combination with cytarabine, followed by oral mercaptopurine, oral methotrexate and oral ATRA for >2 years as maintenance therapy. Our study was authorized by the Ethical Committee and Institutional Review Board of the Affiliated People’s Hospital of Jiangsu University (Zhenjiang, China). Patients and families were required to sign the informed consent permitting that their BM samples would be utilized for scientific research on the principle of voluntary participation.

### Public datasets

In total, three publicly available AML datasets from The Cancer Genome Atlas (TCGA) and Gene Expression Omnibus (GEO) (https://www.ncbi.nlm.nih.gov/geo/) were used to validate our results in this study. Of these data sets, two consisted of expression data for large primary AML samples (GSE12417 and the TCGA dataset) and one dataset contained both AML and healthy BM samples sorted by fluorescence-activated cell sorting (GSE63270). GSE42519, which includes gene expression data of normal hematopoietic cells, was used to explore the expression pattern of DLEU7-AS1 at various stages of normal hematopoiesis. The Genotype-Tissue Expression (GTEx; https://www.GTExportal.org/home/) and the Cancer Cell Line Encyclopedia (CCLE; https://www.broadinstitute.org/ccle) databases were utilized to assess the expression of DLEU7-AS1 in different human tissues and cancer cell lines.

### Reverse transcription-quantitative PCR (RT-qPCR)

The total RNA was separated from pre-extracted BMMNCs using TRIzol® reagent (Invitrogen, Carlsbad, CA, U.S.A.) and then reverse transcribed (MBIFermentas, Hanover, MD, U.S.A.) into complementary DNA (cDNA) in an iCycler Thermal Cycler (Eppendorf, Hamburg, Germany) as described in our previous article [18]. Subsequently, a 7500 Thermo cycler (Applied Biosystems, CA, U.S.A.) was utilized to perform qPCR, with each reaction system containing 20 μl, consisting of 0.4 μM ROX Reference Dye II (Takara Bio, Inc., Tokyo, Japan), 0.8 μM primers, 10 μM SYBR Premix TB Green and 20 ng cDNA. The thermocycling conditions for the qPCR reaction were 95°C for 5 min, followed by 40 cycles at 95°C for 10 s, 64°C for 30 s, 72°C for 30 s and 80°C to collect fluorescence for 30 s; the final step was 95°C for 15 s, 60°C for 60 s, 95°C for 15 s and 60°C for 15 s. Serial dilution was performed and calibration curves were generated to test for PCR efficiency. Positive controls and negative controls were used in each trial to exclude false negatives and false positives. The abundance of *DLEU7-AS1* was calculated by normalizing to the housekeeping gene *ABL1* [19]. The following primers were used for qPCR: *DLEU7-AS1* reverse, 5′-AGTTCTCCCTTGCTGCACTC-3′ and forward, 5′-TATGGCCGAGCAGACATTGG-3′; ABL1 reverse, 5′-TCCAACGAGCGGCTTCAC-3′ and forward, 5′-TCCTCCAGCTGTTATCTGGAAGA-3′. The results were analyzed using the 7500 Thermo cycler software and the relative expression levels of *DLEU7-AS1* were calculated using the 2^−ΔΔCq^ method [19].

### Gene mutation detection

*Isocitrate dehydrogenase 1/2*, *C-KIT*, *NPM1*, *DNA methyltransferase 3A*, *U2 small nuclear RNA auxiliary factor* and *N/K-RAS* mutations were detected using high-resolution melting analysis [20–24], as this method was demonstrated by our group to have high sensitivity and specificity for assessing these gene mutations. *FLT3*-ITD and *CEBPA* mutations were detected by RT-qPCR [25,26]. Finally, direct DNA sequencing was used to verify all the positive samples (BGI Tech Solutions Co, Shanghai, China).

### Statistical analyzes and bioinformatics

Data analyses were performed using the IBM SPSS Statistics 25.0 software (IBM Corporation, Armonk, NY, U.S.A.). The expression values were first log_2_ transformed before the differences in expression were calculated using the Wilcoxon rank-sum test. A receiver operating characteristic curve (ROC) was utilized to evaluate if DLEU7-AS1 expression could be used to distinguish patients with AML from healthy individuals. A Fisher’s exact test or Pearson's χ^2^ analysis was applied to evaluate the association between each of the classification variables and patients in the DLEU7-AS1^high^ expression group or those in the DLEU7-AS1^low^ group. The effect of DLEU7-AS1 expression on the survival time of patients with AML was analyzed using the Kaplan–Meier method and Cox regression analysis, with the optimal cut-off point determined using X-tile analysis. The correlation between DLEU7-AS1 and DLEU7 expression was analyzed through a Pearson’s correlation test. Correlation between the expression of DLEU7-AS1 with immunocyte infiltration and immune checkpoint genes in the TCGA cohort were assessed using the Sangerbox software 3.0 (https://sangerbox.com/). Differential gene expression analysis was performed using the limma R package. Differentially expressed genes (DEGs) between patients with high and low DLEU7-AS1 expression were illustrated using a volcano plot using the “EnhancedVolcano” R package. Genes with an adjusted *P*-value <0.05 and |log_2_ fold change| ≥0.5 were considered to be DEGs. STRING database version 11.5 (https://www.string-db.org/) was applied to analyze the protein-protein interactions (PPI) and Gene Ontology (GO) enrichment of *DLEU7-AS1* co-expressed genes. For PPI analysis, a confidence score >0.9 was considered to be the judgment criterion. Cytoscape version 3.8.2 was used to present the network. All graphs were produced using GraphPad Prism® 7.0 (GraphPad Software, San Diego, California) and R version 4.0.3 (https://www.r-project.org/). *P*<0.05 was applied as the threshold of statistical significance in all tests.

## Results

### DLEU7-AS1 expression in normal human tissues and cancer cell lines

**Figure 1:**
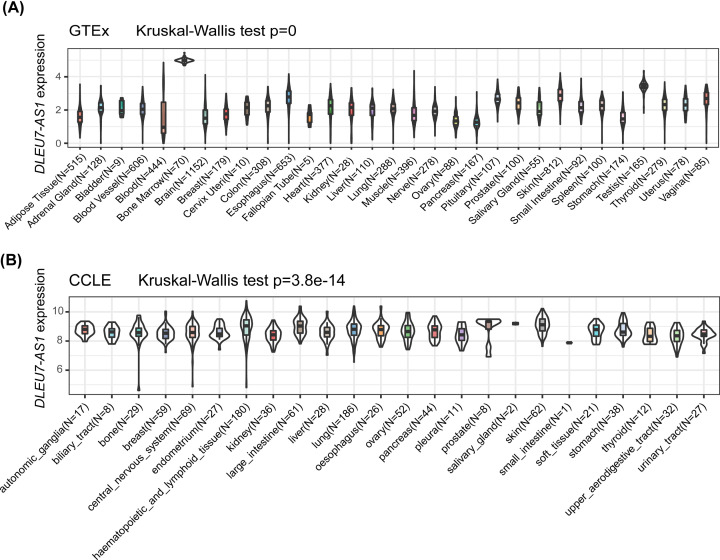
The expression levels of *DLEU7-AS1* in tissues of differing origin and different cancer cell lines (**A**) mRNA expression of *DLEU7-AS1* in different tissues according to data obtained from the GTEx database. *DLEU7-AS1* showed the highest expression in the bone marrow. (**B**) mRNA expression of *DLEU7-AS1* in various tumor cell lines from the CCLE database. *DLEU7-AS1* showed the highest expression in the hematopoietic cancer cell lines.

Analysis of transcriptional data from GTEx (http://www.GTExportal.org/home/) revealed that *DLEU7-AS1* expression was the highest in the bone marrow (Figure 1A). Furthermore, CCLE database analysis identified relatively high levels of *DLEU7-AS1* mRNA expression in the hematopoietic cell lines (Figure 1B). It should be noted that *DLEU7-AS1* expression across all tissues was fairly even and the high expression observed in hematopoietic tissue is probably a reflection of the larger expression variation across this tissue dataset.

### *DLEU7-AS1* expression levels in patients with AML and normal controls

**Figure 2:**
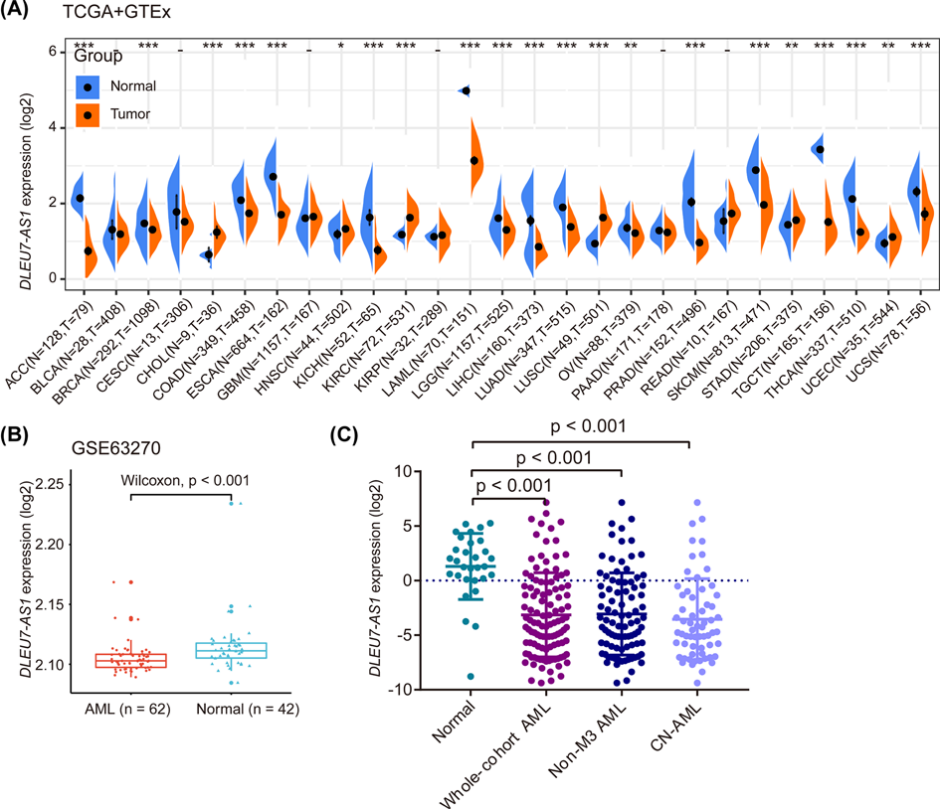
The expression levels of DLEU7-AS1 in tissues of differing origin and different cancer cell lines (**A**) Comparison of *DLEU7-AS1* mRNA expression among normal, peri-tumor and tumor samples, using combined data from the TCGA and GTEx databases. The GTEx data profiled RNAseq samples from normal bone marrow, which were used as normal controls of TCGA AML samples. * denotes expression of this gene in one sample. (**B**) Box plots showing the *DLEU7-AS1* expression levels in healthy individuals and patients with AML using the GSE63270 published dataset (*n*=104). (**C**) The levels of *DLEU7-AS1* expression of in healthy individuals, whole-cohort patients with AML, patients with non-M3 AML, and patients with CN-AML as measured using RT-qPCR. The distribution of log_2_-transformed *DLEU7-AS1* expression in healthy individuals, whole-cohort patients with AML, patients with non-M3 AML and patients with CN-AML are presented as scatter plots. The mean and SD level of *DLEU7-AS1* expression in each group are presented with a horizontal line.

**Figure 3:**
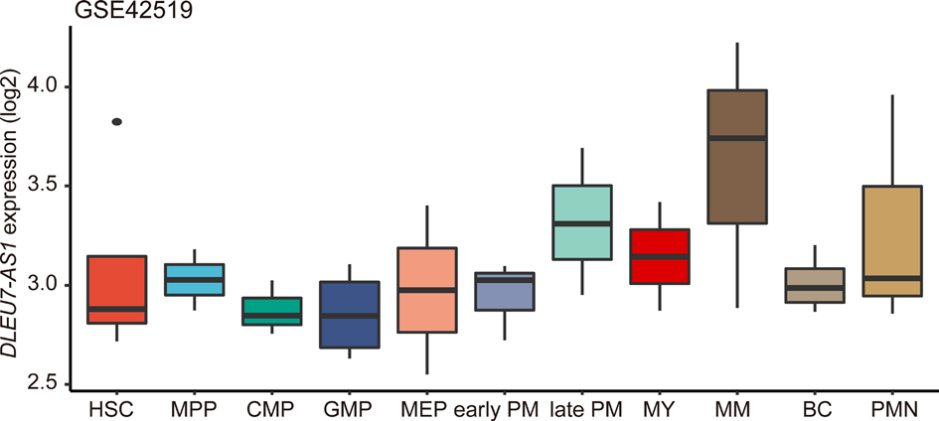
Expression of *DLEU7-AS1* at multiple stages of hematopoietic cell differentiation using the GSE42519 dataset *DLEU7-AS1* expression was relatively low in the early stages but increased gradually in more differentiated blood cells from normal individuals.

We then integrated normal tissue transcription data from GTEx and tumor-adjacent normal tissue data from TCGA to assess if *DLEU7-AS1* expression was dysregulated in different types of cancer. Although *DLEU7-AS1* expression was found in AML among all malignancies analyzed, its expression was found to be markedly decreased in AML compared with that in healthy controls (Figure 2A). In addition, decreased *DLEU7-AS1* expression was also observed in AML samples in an independent GEO dataset (GSE63270; *P*<0.001) (Figure 2B). To verify these findings, we detected *DLEU7-AS1* expression in 110 patients with newly diagnosed AML and 30 normal controls using RT-qPCR as previously described [19]. The expression level of *DLEU7-AS1* in the normal control group was between 0.002 and 38.349 (median, 2.953). However, the level of *DLEU7-AS1* transcripts in patients with AML was between 0.015 and 143.529 (median, 0.068), which was found to be significantly lower compared with that in the normal control group according to a non-parametric test (*P*<0.001) (Figure 2C). *DLEU7-AS1* expression was also found to be significantly lower in both non-M3 and CN-AML subgroups as compared with normal controls (Figure 2C). To validate these findings and explore the expression pattern of *DLEU7-AS1* during the different stages of normal hematopoiesis, the level of *DLEU7-AS1* transcripts was analyzed using another published GEO dataset (GSE42519). We found relatively low *DLEU7-AS1* expression during the early stages of hematopoietic differentiation, but the quantity of this transcript was increased as the cell lineages matured, with the highest expression level observed in metamyelocytes (Figure 3).

### Distinguishing capacity of using differential *DLEU7-AS1* expression levels

**Figure 4:**
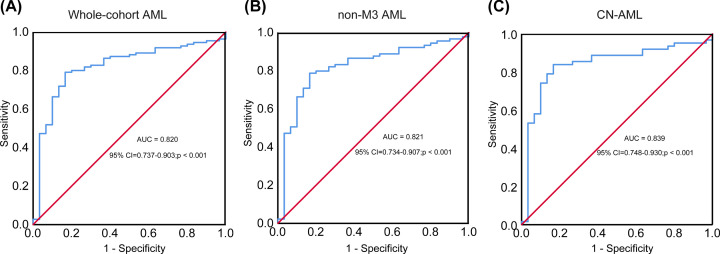
Expression of DLEU7-AS1 at multiple stages of hematopoietic cell differentiation using the GSE42519 dataset The area under the curve values for (**A**) whole-cohort patients with AML, (**B**) for patients with non-M3 AML and (**C**) for patients with CN-AML, which were calculated to be 0.820 (95% CI: 0.737–0.903; *P*<0.001), 0.821 (95% CI: 0.734–0.907; *P*<0.001) and 0.839 (95% CI: 0.748–0.930; *P*<0.001), respectively.

ROC curve analysis revealed that differential *DLEU7-AS1* expression levels could be used to distinguish whole cohorts of patients with AML from the normal control group (Figure 4A). The area under the curve (AUC) was 0.820 (95% CI: 0.737–0.903; *P*<0.001). Using the cut-off value of <0.089, the specificity and sensitivity for discriminating AML were calculated to be 90 and 55%, respectively. In addition, significant differences were also observed in non-M3 AML subgroup of patients (AUC = 0.821; 95% CI: 0.734–0.907; *P*<0.001) (Figure 4B) and the CN-AML subgroup of patients (AUC = 0.839; 95% CI: 0.748–0.930; *P*<0.001) (Figure 4C).

### Comparison of clinical parameters between *DLEU7-AS1*^low^ and *DLEU7-AS1*^high^ groups

**Figure 5:**
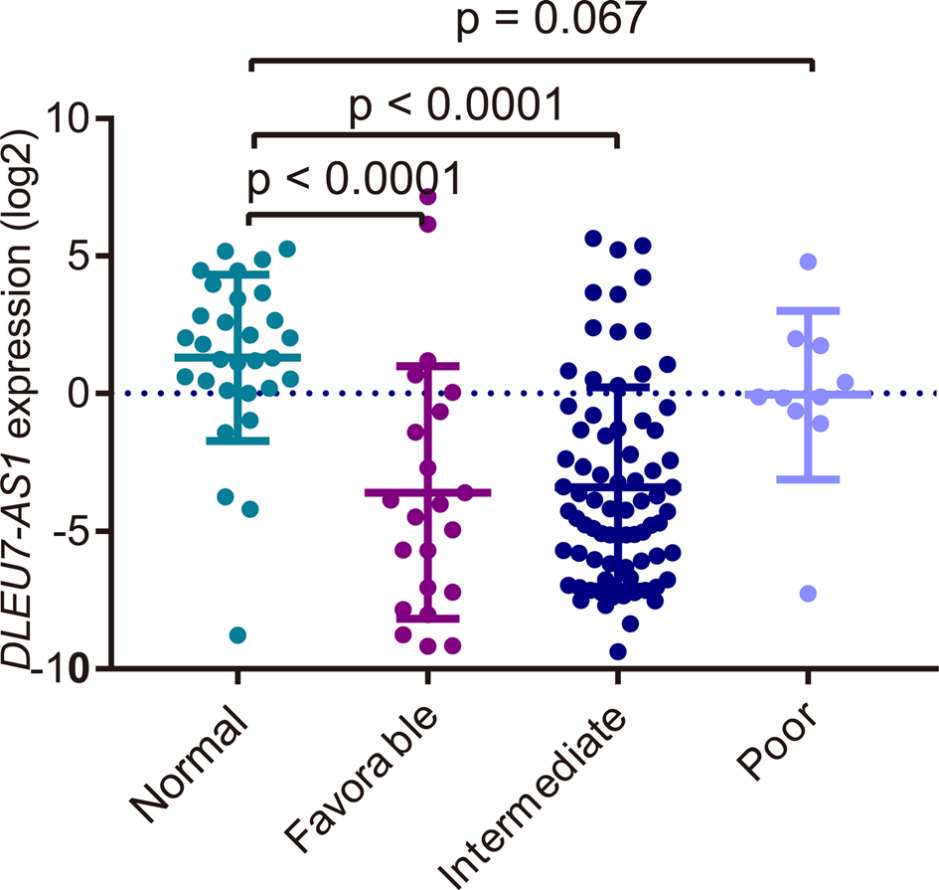
Relative expression levels of *DLEU7-AS1* in healthy individuals and different karyotypic risk stratification groups of patients with AML The distributions of log_2_-transformed *DLEU7-AS1* expression in controls and patients with AML with favorable karyotypes, intermediate karyotypes and poor karyotypes are presented in the scatter plots. The mean ± SD expression of *DLEU7-AS1* in each patient group is presented with horizontal lines.

We divided the entire group of patients with AML from our cohort (*n*=110) into the *DLEU7-AS1*^low^ and *DLEU7-AS1*^high^ groups according to the cut-off value of 0.089 based on the ROC curve. A comparison of the clinical parameters of the two groups in the respective available data are shown in Table 1. We observed no significant difference in age, white blood cell (WBC) count, sex distribution, BM blasts ratio and hemoglobin levels between the two groups of patients (*P*>0.05). There were also no statistically significant differences in the distribution of WHO classifications or in the FAB subtypes between these two groups (*P*>0.05). However, the *DLEU7-AS1*^low^ group had significantly lower baseline platelet counts compared with those in the *DLEU7-AS1*^high^ group (*P*=0.029). In addition, a significant difference was found between the *DLEU7-AS1*^low^ and *DLEU7-AS1*^high^ groups in the distribution of karyotype classification (*P*=0.008). The frequency of favorable karyotype (65%, 15/23) and intermediate karyotype (58%, 44/76) was significantly higher compared with that of the poor karyotype (10%, 1/10) in the *DLEU7-AS1*^low^ group (*P*=0.005). The expression levels of *DLEU7-AS1* in patients with favorable (median, 0.053) and intermediate karyotypes (median, 0.053) were significantly decreased compared with those in the normal group (*P*<0.0001). By contrast, those with poor karyotypes (median, 0.915) had a trend toward lower *DLEU7-AS1* expression compared with that in the normal control group (*P*=0.067) (Figure 5). However, there was no significant correlation between the frequencies of common gene mutations (such as CEBPA, NPM1, and FLT3-ITD) and *DLEU7-AS1* expression (*P*>0.05) (Table 1).

**Table 1:** Comparison of clinical parameters between AML patients with low and high *DLEU7-AS1* expression

Patient’s parameters	High (*n*=50)	Low (*n*=60)	*P* value
Sex, male/female	33/17	33/27	0.241
Median age, years (range)	60.50 (15-81)	53.50 (10-84)	0.074
Median WBC, ×10^9^/L (range)	9.20 (3-528)	10.30 (0.8-207.9)	0.335
Median platelets, ×10^9^/L (range)	50.00 (7-415)	30.00 (3-215)	0.029
Median hemoglobin, g/L (range)	76.50 (34-138)	78.50 (36-144)	0.358
BM blasts, % (range)	43.0 (1.0-97.5)	45.25 (3.0-95.0)	0.692
FAB			0.420
M0	0	1	
M1	1	1	
M2	19	21	
M3	8	13	
M4	12	10	
M5	6	4	
M6	1	0	
Unclassified	3	10	
Karyotype classification			0.008
Favorable	8 (16.0%)	15 (25.0%)	
Intermediate	32 (64.0%)	44 (73.3%)	
Poor	9 (18.0%)	1 (1.7%)	
No data	1 (2%)	0 (0%)	
Karyotype			0.140
Normal	24 (48.0%)	38 (63.3%)	
t (8;21)	2 (4.0%)	3 (5.0%)	
t (15;17)	8 (16.0%)	11 (18.3%)	
t (9;22)	0 (0%)	1 (1.7%)	
+8	2 (4.0%)	1 (1.7%)	
complex	7 (12.0%)	1 (1.7%)	
others	6 (10.0%)	5 (8.3%)	
No data	1 (6.0%)	0 (0%)	
Gene mutation			
*CEBPA* (+/-)	6/25	5/40	0.340
*NPM1* (+/-)	3/28	6/39	0.730
*FLT3*-ITD (+/-)	4/27	6/38	1.000
*C-KIT*(+/-)	1/30	2/43	1.000
*N/K-RAS* (+/-)	3/28	2/36	0.651
*IDH1/2* (+/-)	0/31	1/42	1.000
*DNMT3A* (+/-)	3/28	3/42	0.683
*U2AF1* (+/-)	2/29	0/45	0.163
CR (-/+)	34/12	23/30	0.002

Abbreviations: AML/M, acute myeloid leukemia; BM, bone marrow; CR, complete remission; FAB: French-American-British; t, translocation; WBC, white blood cell.

### Association between *DLEU7-AS1* expression and clinical outcomes in patients with *de novo* AML

**Figure 6:**
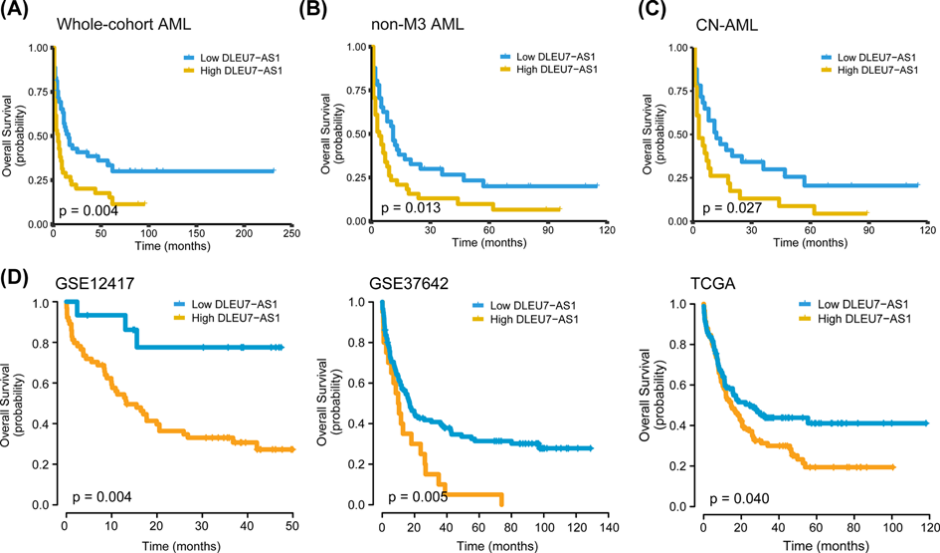
Investigation of using *DLEU7-AS1* expression as a potential predictor of AML outcome (**A–C**) Kaplan–Meier estimate for the OS of the Chinese cohort of patients in the whole-cohort (**A**), non-M3 (**B**) and (**C**) CN-AML group. (**D**) Validation of the prognostic value of using *DLEU7-AS1* expression in three independent cohorts of patients with AML (GSE12417, *n*=79; GSE37642, *n*=136; TCGA microarray, *n*=183). Survival analyzes were performed on the *DLEU7-AS1^low^* and *DLEU7-AS1^high^* groups and *P*-values were calculated using the Kaplan–Meier method.

Next, the relationship between *DLEU7-AS1* expression and outcome was analyzed in a total of 99 patients (our cohort) who had available follow-up data. Patients in the *DLEU7-AS1*^low^ group had significantly higher complete response (CR) rates compared with those in the *DLEU7-AS1*^high^ group (57 vs. 26%; *P*=0.002) (Table 1). In addition, the *DLEU7-AS1*^low^ group also had significantly higher CR rates compared with those in the *DLEU7-AS1*^high^ group in the non-M3 subgroup [46%, (19/41) vs. 20% (8/40); *P*=0.012]. The difference in the CR rate between patients in the *DLEU7-AS1*^low^ and *DLEU7-AS1*^high^ groups was not significant in the CN-AML subgroup [44% (14/32) vs. 22% (5/23); *P*=0.09]. In addition, survival analysis revealed that patients in the *DLEU7-AS1*^low^ group exhibited superior overall survival (OS) rates (*P*=0.004) (Figure 6A) compared with those in the *DLEU7-AS1*^high^ group in the whole AML cohort. However, the impact of *DLEU7-AS1* expression on leukemia-free survival was not statistically significant (data not shown). We also tested the prognostic value of *DLEU7-AS1* expression in the non-M3 (*P*=0.013) (Figure 6B) and CN-AML subgroups (*P*=0.027) (Figure 6C), where it was found to be significantly associated with longer OS. Overall, these results suggest that *DLEU7-AS1* expression can be concluded to be a viable prognostic predictor in both the whole cohort in addition to the non-M3 or CN-AML subgroups. Subsequently, the prognostic value of *DLEU7-AS1* expression for OS was also validated in one CN-AML cohort from the GSE12417 dataset (*n*=79; *P*=0.004) and the dataset from two large primary AML cohorts (GSE37642, *n*=136, *P*=0.005; TCGA microarray, *n*=183, *P*=0.040) (Figure 6D), where low *DLEU7-AS1* expression remained an indicator of favorable OS.

Finally, common clinical parameters, including the cytogenetic risk group (favorable vs. intermediate vs. poor), WBC counting (≥30 × 10^9^/L vs. <30 × 10^9^/L), age (≤60 vs. >60 years), *DLEU7-AS1* expression status and molecular mutations in our cohort were analyzed using univariate Cox models. Variables with *P*<0.2 in univariate analysis were further included in a multivariate model. However, multivariate analysis failed to reveal the independent prognostic significance of *DLEU7-AS1* expression in patients with CN-AML (data not shown), whole-cohort AML (data not shown) or non-M3 (Table 2).

**Table 2:** Univariate and multivariate analysis of variables for overall survival in non-M3 AML patients

Variables	Overall survival			
	Univariate analysis		Multivariate analysis	
	HR (95% CI)	*P* value	HR (95% CI)	*P* value
WBC	1.891 (1.163–3.075)	0.010	1.524 (0.913–2.543)	0.107
Age	2.252 (1.367–3.709)	0.001	2.252 (1.367–3.709)	0.001
*DLEU7-AS1* expression	1.786 (1.098–2.906)	0.019	1.502 (0.907–2.488)	0.114
Karyotype risk	1.439 (0.914–2.264)	0.116	1.248 (0.771–2.020)	0.368
*FLT3*-ITD mutation	1.348 (0.598–3.040)	0.471	–	–
*NPM1* mutation	1.146 (0.510–2.574)	0.742	–	–
*CEBPA* mutation	0.649 (0.274–1.540)	0.327	–	–
*c-KIT* mutation	0.367 (0.050–2.681)	0.323	–	–
*N/K-RAS* mutation	0.429 (0.103–1.793)	0.246	–	–
*IDH1/2* mutation	1.938 (0.457–8.215)	0.369	–	–
*DNMT3A* mutation	1.216 (0.477–3.100)	0.682	–	–

**Notes:** Variables including, WBC (≥30 × 10^9^ vs. <30 × 10^9^ /L), age (≤60 vs. >60 years), *DLEU7-AS1* expression (low vs. high), karyotype risk (favorable vs. intermediate vs. poor) and gene mutations (mutant vs. wild-type). Multivariate analysis includes variables with *P*<0.200 in univariate analysis.

**Abbreviations:** AML, acute myeloid leukemia; CI, confidence interval; HR, hazard ratio; WBC, white blood cell.

We deemed that there may be other stronger prognostic indicators. As shown in Table 2, we observed that age was an important independent risk factor in the non-M3 subgroup. Subsequently, patients who were aged ≤60 years old were separated for further study. A In this subgroup analysis, significant difference was found in the OS of patients in the *DLEU7-AS1*^low^ and *DLEU7-AS1*^high^ groups patients according to univariate analysis (*P*=0.047) (Table 3). Multivariate Cox analysis indicated that *DLEU7-AS1* expression was also an important prognostic indicator [HR = 2.146; 95% CI (1.023–4.501); *P*=0.043] (Table 3) in this subset.

**Table 3:** Univariate and multivariate analysis of variables for overall survival in non-M3 AML patients who are less than or equal 60 years old

Variables	Overall survival			
	Univariate analysis		Multivariate analysis	
	HR (95% CI)	*P* value	HR (95% CI)	*P* value
WBC	2.381 (1.111–5.100)	0.026	2.429 (1.127–5.236)	0.024
*DLEU7-AS1* expression	2.106 (1.009–4.397)	0.047	2.146 (1.023–4.501)	0.043
Karyotype risk	1.695 (0.800–3.590)	0.168	1.522 (0.668–3.468)	0.317
*FLT3*-ITD mutation	1.787 (0.406–7.858)	0.442	–	–
*NPM1* mutation	1.418 (0.522–3.852)	0.493	–	–
*CEBPA* mutation	0.529 (0.156–1.796)	0.307	–	–
*N/K-RAS* mutation	0.330 (0.044–2.475)	0.281	–	–
*DNMT3A* mutation	0.957 (0.223–4.118)	0.953	–	–

**Notes:** Variables including, WBC (≥30 × 10^9^ vs. <30 × 10^9^ /L), age (≤60 vs. >60 years), *DLEU7-AS1* expression (low vs. high), karyotype risk (favorable vs. intermediate vs. poor) and gene mutations (mutant vs. wild-type). Multivariate analysis includes variables with *P*<0.200 in univariate analysis.

**Abbreviations:** AML, acute myeloid leukemia; CI, confidence interval; HR, hazard ratio; WBC, white blood cell.

### Correlation between the expression levels of *DLEU7-AS1* and *DLEU7*

**Figure 7:**
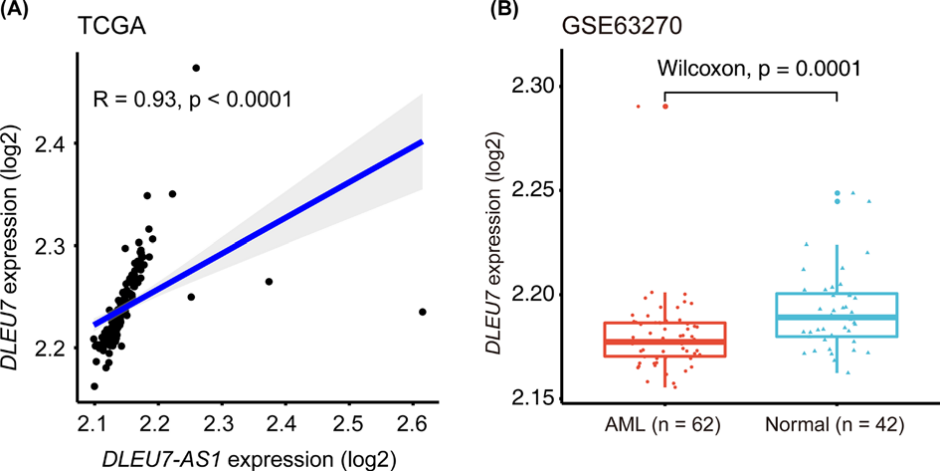
Correlation between the expression levels of *DLEU7-AS1* and DLEU7 (**A**) Pearson’s correlation analysis between *DLEU7-AS1* and DLEU7 expression in patients with primary AML (TCGA microarray, *n*=183). (**B**) Box plots showing DLEU7 expression in healthy individuals and patients with AML using a published dataset (GSE63270, *n*=104).

Since *DLEU7-AS1* is the non-coding strand of the *DLEU7* gene, we hypothesized that their expression might be correlated. To explore the relationship between the expression of *DLEU7-AS1* and DLEU7, an independent cohort containing the expression information of 183 patients with *de novo* AML derived from TCGA was included in this study. Pearson’s correlation analysis revealed that *DLEU7-AS1* and DLEU7 expression were positively correlated (*R* = 0.93; *P*<0.0001) (Figure 7A). Furthermore, the quantity of *DLEU7* transcripts was significantly decreased in AML samples compared with that in normal controls (GSE63270; *P*=0.0001) (Figure 7B).

### Association between the expression levels of *DLEU7-AS1* with immunocyte infiltration and immune checkpoint genes in AML

**Figure 8:**
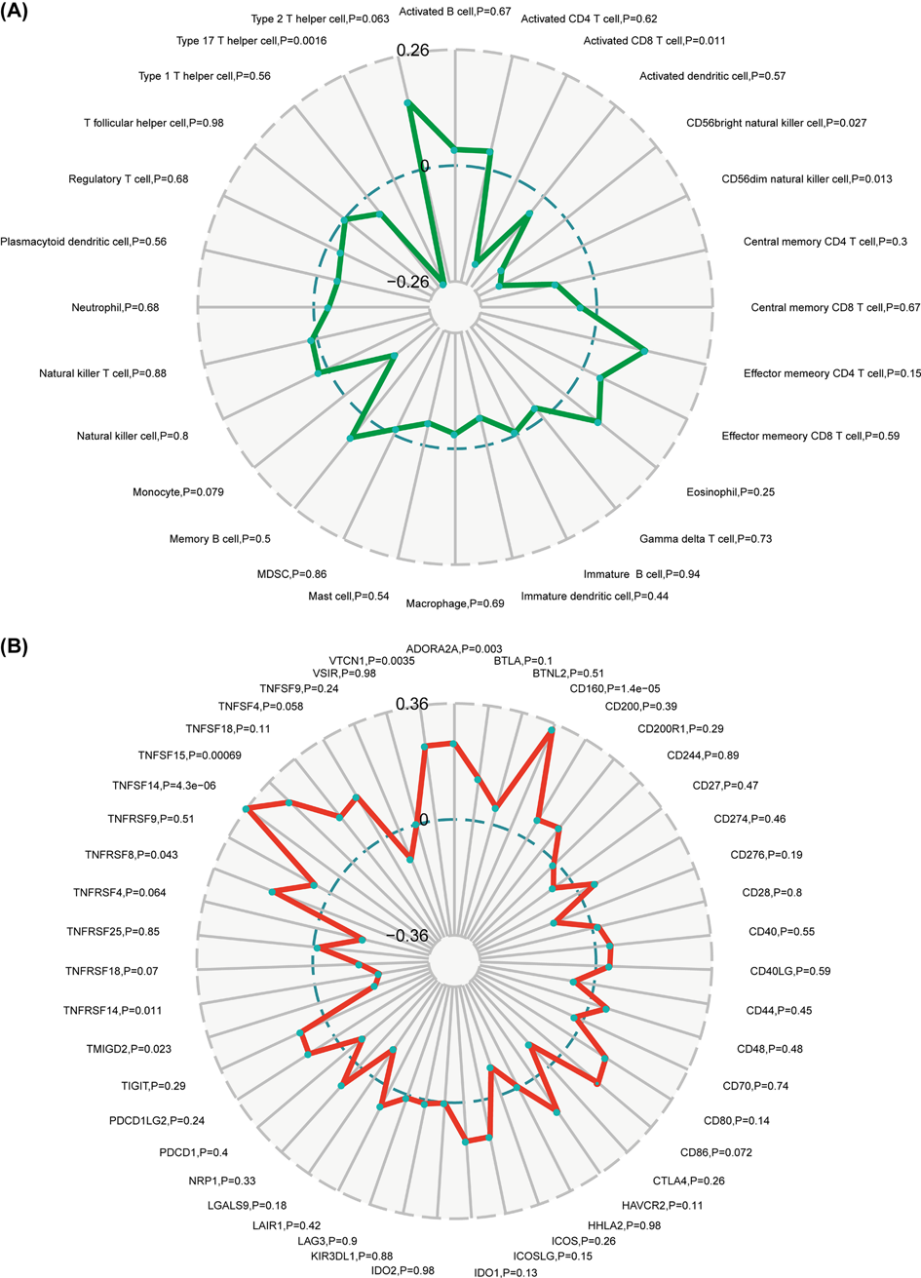
Correlation between the expression levels of *DLEU7-AS1* with the immune component of the TCGA LAML cohort (**A**) Immunocyte infiltration. (**B**) Immune checkpoint genes.

Next, we investigated the possible correlation between *DLEU7-AS1* expression and immunocyte infiltration levels using CIBERSORT in the TCGA AML cohort. We found *DLEU7-AS1* expression to be negatively correlated with tumor-suppressive components, such as activated CD8 T cells and natural killer cells (Figure 8A). The most significant negative association was observed for type 17 T helper cells (*P*=0.0016), whilst monocytes (*P*=0.079) and type 2 T helper cells (*P*=0.063) also showed a trend toward significant association with *DLEU7-AS1* expression (Figure 8A).

Subsequently, we analyzed the association between *DLEU7-AS1* expression and checkpoint gene expression. We found a positive association between *DLEU7-AS1* expression with *V-Set domain-containing T Cell activation inhibitor 1*, *adenosine A2A receptor*, *CD160* and tumor necrosis factor (TNF)-related immune genes (*TNFRSF8*, *TNFRSF14*, *TNFSF14* and *TNFSF15*), whilst the expression of *DLEU7-AS1* was negatively correlated with *transmembrane and immunoglobulin domain-containing 1 (TMIGD1), HERV-H LTR-associating 2* (HHLA2; a receptor of the B7 family member). TMIGD1 and HHLA2 were previously reported to be potential therapeutic targets in cancers [27] (Figure 8B).

### Associations between genome-wide gene expression profiles and *DLEU7-AS1* expression

**Figure 9:**
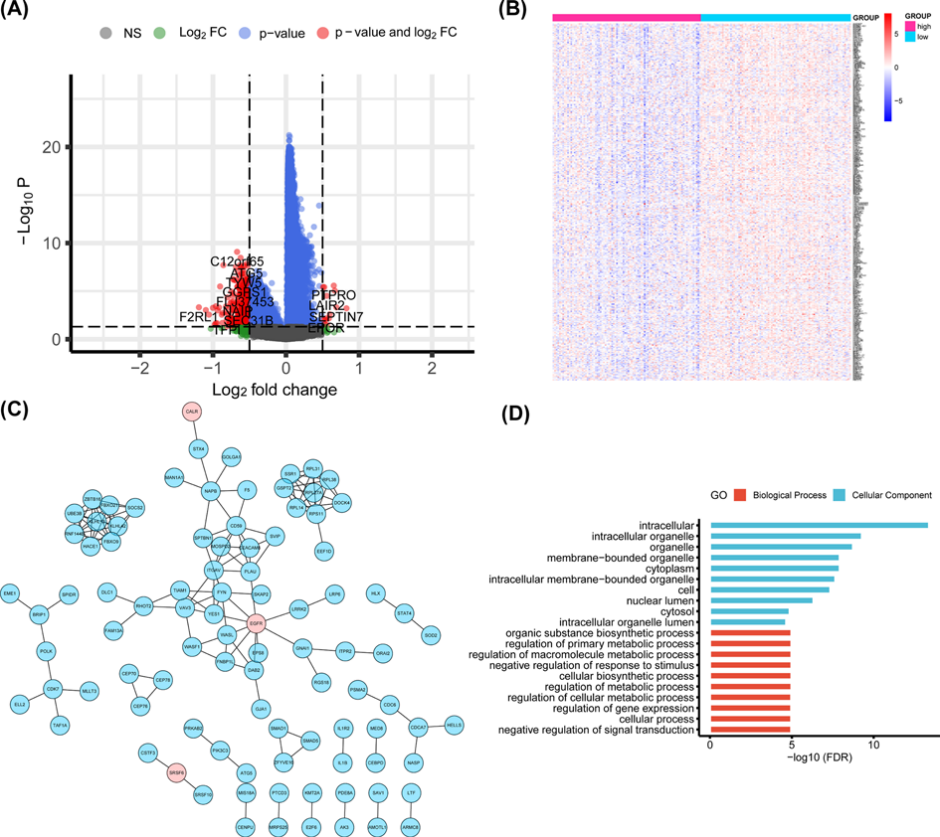
Associations between genome-wide gene-expression profiles and *DLEU7-AS1* expression (**A**) Volcano plot and (**B**) heatmap of DEGs between high and low levels of *DLEU7-AS1* expression in the TCGA cohort. (**C**) PPI network of these DEGs. The nodes denote proteins whereas the edges denote the interactions of proteins. Blue points indicate down-regulated genes whilst red points indicate up-regulated genes. (**D**) GO analysis of *DLEU7-AS1* and its co-expressed genes.

To further assess the role of *DLEU7-AS1* in AML, we derived *DLEU7-AS1*-associated gene expression profiles using the TCGA microarray data (*n*=183). We identified 14 up-regulated and 373 down-regulated genes that were found to be significantly associated with *DLEU7-AS1* expression (adjusted *P*<0.05; |log_2_ fold change| >0.5) (Figure 9A,B). Subsequently, we constructed a PPI network using the DEGs by STRING to explore potential interactions among them. In this network, we noticed that the expression of one potential therapeutic target, EGFR, was up-regulated in the *DLEU7-AS1*^high^ group, where it directly interacted with *WASP-like actin nucleation promoting factor*, *formin binding protein 1*, *LGNAI1*, *leucine rich repeat kinase 1*, *FYN*, plasminogen activator, urokinase, *YES1*, *epidermal growth factor receptor pathway substrate 8* and *disabled homolog 2* (Figure 9C). In addition, GO analysis was performed to analyze the potential role of *DLEU7-AS1* and the co-expressed genes. We found that several metabolism-related biological processes, such as regulation of primary metabolic process, regulation of macromolecule metabolic process, regulation of metabolic process and regulation of cellular metabolic process were enriched (Figure 9D). Moreover, cellular components, including intracellular, intracellular organelle, organelle, membrane-bounded organelle, cytoplasm, intracellular membrane-bounded organelle, cell, nuclear lumen, cytosol and intracellular organelle lumen, were significantly associated with the *DLEU7-AS1* alteration (Figure 9D). However, no significant enrichment in molecular functions or pathways were found for these DEGs.

## Discussion

*DLEU7-AS1* is an antisense lncRNA that was previously found to be uniquely expressed in specific cancer types or differentiated tissues [28]. Antisense lncRNAs are a specific type of noncoding RNA that can regulate gene expression in the nucleus and cytoplasm through RNA–DNA interactions, transcription interference or RNA–RNA interactions [29]. A number of studies have previously indicated that antisense lncRNAs are also involved in genomic imprinting [30], X-chromosome inactivation [31,32], progression and development of certain diseases [33]. However, the *DLEU7-AS1* expression pattern and its downstream role in hematological malignancies remain unknown. Since negative *DLEU7* expression and hypermethylation of its promoter were previously found in patients with B-cell chronic lymphocytic leukemia (B-CLL), *DLEU7* was previously proposed to be a candidate cancer suppressor gene in CLL located in 13q14 [34]. The most common aberration is 13q deletion, which results in the reduced expression of microRNAs, such as miR-15a and miR16-1 [34,35]. They were also considered to be important in the pathogenesis of CLL and have an influence on TP53 expression and activity [34,35]. These results suggested that *DLEU7-AS1* and *DLEU7* can serve an important role in AML.

In the present study, we first investigated *DLEU7-AS1* expression levels in patients initially diagnosed with AML and then analyzed its clinical impact. The results revealed that *DLEU7-AS1* expression was dramatically down-regulated in patients with AML compared with that in normal controls. Indeed, the BMMNCs from AML and normal samples contain hematopoietic cells of nonmyeloid origin so that the results can be taken in context. It is recommended that positive sorting or lymphocyte depletion was performed prior to sample processing in future works assessing *DLEU7-AS1* expression. Since the treatment outcome of M3 and non-M3 patients are very different, patients with M3 subtype are thought as confounding factors. While the CN-AML are indeed a heterogeneous group of patients with normal karyotypes. Therefore, in most analyses, we have included the whole cohort, the non-M3 and the CN-AML subsets. ROC curve analysis also revealed that differential *DLEU7-AS1* expression could be used to distinguish patients with whole AML, CN-AML and non-M3 from healthy controls.

During analysis of the expression pattern of *DLEU7-AS1* in the different karyotypic classifications, we revealed that *DLEU7-AS1* expression was markedly reduced in patients with AML with favorable/intermediate karyotypes, but not in patients with poor karyotypes. This seemingly contradictory result may be caused by the relatively small size of our cohort, especially patients with poor karyotypes (*n*=10). In addition, since all patients in this study were selected retrospectively, a potential bias relating to the unbalanced clinical features with treatment heterogeneity cannot be ruled out. Finally, since patients with low *DLEU7-AS1* expression generally had favorable prognosis, this may explain the decreased *DLEU7-AS1* expression in patients with favorable karyotypes. Further prospective studies with larger sample sizes are required to validate this result.

Combining our investigation using Kaplan–Meier analysis together with multivariate analysis, we concluded that the patient’s age and WBC count may have had a stronger influence on the prognosis than *DLEU7-AS1* expression. *DLEU7-AS1* repression during leukemogenesis may depend on the context of favorable and intermediate karyotypes, which may also act as a potential prognostic factor for longer OS in AML. This can add an additional prognostic parameter for stratifying the patients. Additionally, deletion of 13q and the expression of miR-15a and miR16-1 may also be used to explain the differences in OS.

Although increased *DLEU7-AS1* expression has only been reported in CRC, which correlated positively with the Wnt/β-catenin pathway [13]. This suggests that the higher the *DLEU7-AS1* expression level, the poorer the outcome [13]. Our observation in AML, which is consistent with this published data with CRC, suggests that the *DLEU7-AS1* expression profile and its role in tumorigenesis and development may be tissue-specific. In fact, dysregulation of Wnt/β-catenin signaling was previously found to be necessary for leukemia stem cell self-renewal and survival [36] and associated with AML initiation and progression [37]. We speculate that reduced *DLEU7-AS1* expression may inhibit Wnt/β-catenin signaling through conserved binding sites in the favorable/intermediate karyotypes of patients with AML. To support this hypothesis, we performed correlation analyses between *DLEU7-AS1* expression and 117 Wnt pathway genes retrieved from GSEA (https://www.gsea-msigdb.org/gsea/msigdb/cards/KEGG_WNT_SIGNALING_PATHWAY) using the TCGA dataset. The results favorably agreed with the reported role of DLEU7-AS1 in Wnt pathway. We found that *DLEU7-AS1* was significantly associated with Wnt target genes such as *WNT1*, *SOX17* and *SFRP4* (*P*<0.05, |*r*| > 0.3; Supplementary Data S1). However, the positive association between the expression *of DLEU7-AS1* with *DLEU7* and Wnt target gens warrants further experimental verification, in addition to the regulatory relationship between *DLEU7-AS1* and *DLEU7* expression in AML. Functional follow ups and prospective validations will be necessary to further interpret the mechanisms of altered *DLEU7-AS1* expression in AML. It is a promising observation that conserved binding sites for a number of transcription factors can be found in the *DLEU7* promoter, several of which have been reported to serve significant roles in the tissue-specific expression of genes upstream of cell differentiation and proliferation [34]. However, to the best of our knowledge this has not been investigated in other cancer types. Therefore, the underlying pathological roles of *DLEU7-AS1* in different malignancies remain largely unknown and require further study.

In conclusion, our study found that decreased *DLEU7-AS1* expression was associated with favorable/intermediate karyotypes. In addition, in patients with *de novo* AML, downregulation of *DLEU7-AS1* expression may serve as a potentially viable prognostic indicator.

## Data Availability

The datasets analyzed in this study are available in the following open access repositories: GTEx, www.gtexportal.org/; CCLE, https://www.broadinstitute.org/ccle; TCGA, https://portal.gdc.cancer.gov/, http://www.cbioportal.org; GEO, https://www.ncbi.nlm.nih.gov/geo/ (GEO accession numbers: GSE63270, GSE42519, GSE37642, and GSE12417); CIBERSORT, https://cibersort.stanford.edu/. Other inhouse data used and/or analyzed during the current study are available from the corresponding author on reasonable request.

## Abbreviations

AML: acute myeloid leukemia
ATRA: all-trans retinoic acid
BAALC: brain and acute leukemia cytoplasmic protein
BM: bone marrow
BMMNC: BM mononuclear cell
CCLE: Cancer Cell Line Encyclopedia
cDNA: complementary DNA
CEBPA: CCAAT/enhancer-binding protein α
CN-AML: cytogenetically normal AML
CR: complete remission
CRC: colorectal cancer
DEG: differentially expressed gene
*DLEU7-AS1*: deleted in lymphocytic leukemia 7-antisense 1
*DLEU7-AS1* ^high^: high *DLEU7-AS1* expression
*DLEU7-AS1* ^low^: low *DLEU7-AS1* expression
ELN: European leukemiaNET
ERG: E-26 transformation-specific-related gene
EVI1: Echovirus 11
FAB: French-American-British
FLT3-ITD: fms-like tyrosine kinase 3- internal tandem duplication
GEO: Gene Expression Omnibus
GO: Gene Ontology
GTEx: Genotype-Tissue Expression project
LncRNA: long non-coding RNA
non-M3: non-acute promyelocytic leukemia
NPM1: nucleophosmin 1
OS: overall survival
PPI: protein–protein interactions
ROC: receiver operating characteristic curve
TCGA: The Cancer Genome Atlas
WBC: white blood cell
WHO: World Health Organization
